# Growth response of the ichthyotoxic haptophyte, *Prymnesium parvum* Carter, to changes in sulfate and fluoride concentrations

**DOI:** 10.1371/journal.pone.0223266

**Published:** 2019-09-27

**Authors:** Rakib H. Rashel, Reynaldo Patiño

**Affiliations:** 1 Department of Biological Sciences and Texas Cooperative Fish and Wildlife Research Unit, Texas Tech University, Lubbock, Texas, United States of America; 2 U.S. Geological Survey, Texas Cooperative Fish and Wildlife Research Unit and Departments of Natural Resources Management and Biological Sciences, Texas Tech University, Lubbock, Texas, United States of America; Universidad Nacional Autonoma de Mexico, MEXICO

## Abstract

Golden alga *Prymnesium parvum* Carter is a euryhaline, ichthyotoxic haptophyte (Chromista). Because of its presumed coastal/marine origin where SO_4_^2-^ levels are high, the relatively high SO_4_^2-^ concentration of its brackish inland habitats, and the sensitivity of marine chromists to sulfur deficiency, this study examined whether golden alga growth is sensitive to SO_4_^2-^ concentration. Fluoride is a ubiquitous ion that has been reported at higher levels in golden alga habitat; thus, the influence of F^-^ on growth also was examined. In low-salinity (5 psu) artificial seawater medium, overall growth was SO_4_^2—^dependent up to 1000 mg l^-1^ using MgSO_4_ or Na_2_SO_4_ as source; the influence on growth rate, however, was more evident with MgSO_4_. Transfer from 5 to 30 psu inhibited growth when salinity was raised with NaCl but in the presence of seawater levels of SO_4_^2-^, these effects were fully reversed with MgSO_4_ as source and only partially reversed with Na_2_SO_4_. Growth inhibition was not observed after acute transfer to 30 psu in a commercial sea salt mixture. In 5-psu medium, F^-^ inhibited growth at all concentrations tested. These observations support the hypothesis that spatial differences in SO_4_^2-^ –but not F^-^–concentration help drive the inland distribution and growth of golden alga and also provide physiological relevance to reports of relatively high Mg^2+^ concentrations in golden alga habitat. At high salinity, however, the ability of sulfate to maintain growth under osmotic stress was weak and overshadowed by the importance of Mg^2+^. A mechanistic understanding of growth responses of golden alga to SO_4_^2-^, Mg^2+^ and other ions at environmentally relevant levels and under different salinity scenarios will be necessary to clarify their ecophysiological and evolutionary relevance.

## Introduction

Known in North America as golden alga, *Prymnesium parvum* Carter is a euryhaline, ichthyotoxic haptophyte believed to be of marine or coastal origin but with the ability to grow in estuaries, brackish embayments [1], and inland brackish waters [2,3]. Curiously, however, toxic blooms of this species normally occur within the relatively narrow salinity range of 0.5 to 12 psu (practical salinity unit) [2–4] and abundance is negatively associated with salinity at levels exceeding ~8–12 psu [4–6]. A recent experimental study confirmed the biphasic response of an inland strain of golden alga (UTEX LB 2797) to salinity and reported that the highest growth potential is observed at ~10–15 psu [7]. While multiple abiotic and biotic factors may interact to influence golden alga growth in nature [3,4,8–13], the relatively low salinity range for bloom development seems counterintuitive given its highly euryhaline potential.

The chemical composition of inland surface waters is driven by a complex interaction among the geochemistry of watersheds and aquifers, topography, climate [14], and anthropogenic changes in land cover [15–17]. Little is known about the influence of specific ions on golden alga growth and the role they may play in determining the spatial distribution of this species in inland waters. A retrospective study of reservoir water quality and golden alga in the southcentral USA found that spatial variability in sulfate concentration is associated with the intra- and inter-basin distribution of toxic blooms [11]. Levels of sulfate in reservoirs of the southcentral USA with a history of blooms (average, ~600 mg l^-1^) were >8-fold higher than in naive reservoirs (~70 mg l^-1^) and were nearly equal those of chloride (~800 mg l^-1^) [11]. Blooms of this species in association with high sulfate levels also have been recorded in a few other instances; e.g., in a freshwater pond in Germany [18]. In addition, some studies have reported relatively high levels of fluoride in bloom-impacted (>1 mg l^-1^) compared to non-impacted (<1 mg l^-1^) water bodies of the southcentral USA [12,17]. These findings led to the hypotheses that sulfate [11] and fluoride [12] may influence the distribution and growth of golden alga at least in part via mechanisms independent of salinity.

Sulfate, the primary source of sulfur for algae and plants [19,20], is the second most abundant anion in aquatic habitats after chloride. Its concentration ranges from ~10 to 1300 mg l^-1^ in inland waters [11,12,21] and 2800 to 3000 mg l^-1^ in seawater [22]. Eukaryotic phytoplankton communities in inland waters are typically dominated by green algae [19] but in marine habitats they consist primarily of chromist (chlorophyll a+c) algae, which include the Haptophyta [19,23,24]. Growth of certain green and chromist algae can be reduced under conditions of sulfur deficiency [25–28].

Fluoride is another ubiquitous anion in freshwater with concentration typically ranging from 0.01 to 0.30 mg l^-1^, and in seawater, its concentration ranges from 1.2 to 1.5 mg l^-1^ [29–31]. Considerably higher concentrations have been recorded in inland waters impacted by geothermal or volcanic activity [31]. Fluoride can either inhibit, enhance or not affect algal growth depending upon the species and exposure concentrations [31]. Given the wide variation in responses to fluoride among algal species and the relatively high levels of this ion in golden alga-impacted habitats, VanLandeghem et al. [12] hypothesized that tolerance to fluoride relative to other phytoplankton may confer a growth advantage to golden alga.

The specific objective of this study is to test the hypotheses put forward by earlier field studies that sulfate [11] and fluoride [12] positively influence golden alga growth independently of salinity. While the earlier studies used a multivariate approach to evaluate general associations between environmental variables and golden alga, cause-effect associations could not be addressed because of the complexity of the natural environment and the strong collinearity among several of the variables (e.g., between sulfate and salinity [11]). The present study used an experimental approach where the variables of interest (sulfate and fluoride) were manipulated while maintaining salinity and other ambient conditions constant. To our knowledge, salinity-independent effects of sulfate on growth have not been examined before in inland algae and this study is the first to evaluate salinity-independent effects of sulfate and fluoride on growth of golden alga.

## Materials and methods

### Basic culture procedures

The strain of *P*. *parvum* used in this study, UTEX LB 2797, is the most widespread strain found in US inland waters [32] and was obtained from the UTEX Culture Collection of Algae (The University of Texas at Austin, Texas, USA). Stock cultures were grown in UTEX Artificial Seawater Medium (ASM; https://utex.org/products/artificial-seawater-medium) modified (diluted) to a salinity of 5 and with pH of ~ 8.1. Modified ASM, also referred to as base medium in this study, was enriched with f/2 levels of nutrients and vitamins, and ferrous ammonium sulfate in the original trace metal recipe was replaced with an equimolar amount of ferric chloride [see [7] for additional details of medium preparation]. Cultures were maintained non-axenically in 250-ml Erlenmeyer flasks filled with 100 ml of modified ASM in an incubator (I36LLVL; Percival Scientific Inc. Perry, IA, USA). Temperature and photoperiod were set at 22°C and 12: 12 h light: dark, and light intensity was ~6500 lux. Cultures were gently swirled once daily. Late exponential growth phase cells were used as inocula to maintain stock cultures and as seed for experimental cultures.

### Experimental design

#### Effects of changes in sulfate concentration at low salinity

Earlier field studies reported that inland surface waters with a history of golden alga blooms have relatively high concentrations of sulfate [11,12]. The present experiments were designed to determine whether this association is causal and independent of salinity. Basic culture procedures were the same as for stock cultures. The salinity of modified ASM is 5 psu and its sulfate concentration is 250 mg l^-1^ (Table 1), values which are well within the range observed in golden alga habitat of the Southern Great Plains and the southwestern USA [4,11,12].

**Table 1:** The nominal concentration of major ions or constituents of Artificial Seawater Medium (ASM), Instant Ocean® (IO), and Seawater (SW).

	Major ions (g l^-1^) in experimental media and seawater (SW)						
	Artificial Seawater Medium (ASM)				Instant Ocean (IO)		SW
Ion	5 psu	30 psu	30 psu + MgSO_4_	30 psu + Na_2_SO_4_	5 psu	30 psu	30 psu*
Na^+^	1.77	11.60	11.60	11.60	1.77	10.62	9.27
Mg^2+^	0.06	0.06	0.61	0.06	0.21	1.26	1.10
K^+^	0.08	0.08	0.08	0.08	0.06	0.37	0.34
Ca^2+^	0.02	0.02	0.02	0.02	0.06	0.38	0.35
Cl^-^	2.73	17.90	17.90	17.90	3.08	18.50	16.74
SO_4_^2-^	0.25	0.25	2.40	2.40	0.37	2.21	2.31

*Values are adjusted from concentrations reported for full-strength seawater (35 psu)

In one series of experiments, sulfate concentration was manipulated by replacing MgSO_4_∙7H_2_O (original recipe) with MgCl_2_∙6H_2_O (Sigma M9272) and/or adding different amounts of MgSO_4_∙7H_2_O to achieve concentrations of 0, 50, 250, and 1000 mg l^-1^ while maintaining salinity constant at 5 psu. Salinity of experimental media was confirmed by measurement with a pre-calibrated YSI 85 multiparameter probe (Yellow Springs Incorporated, Yellow Springs, OH, USA). Trace metal compounds containing sulfate in the original recipe were also replaced with alternatives without sulfate; namely, ZnSO_4_∙7H_2_O, MnSO_4_∙H_2_O, and CoSO_4_∙7H_2_O were replaced with equimolar amounts of ZnCl_2_ (Sigma Z0152), MnCl_2_∙4H_2_O (Sigma 221279), and CoCl_2_∙6H_2_O (Sigma 255599), respectively.

Magnesium is a key component of chlorophyll and earlier studies have shown that changes in its concentration can affect algal growth [33]. In a separate experiment, the ability of Mg-free sulfate (as Na_2_SO_4_) to influence golden alga growth was examined. Media with sulfate concentrations of 0, 50, 250, and 1000 mg l^-1^ were prepared by replacing MgSO_4_∙7H_2_O (in original base medium) with, and adding different amounts of, Na_2_SO_4_ (Fisher S421-500) while keeping salinity constant (5 psu). As previously noted, trace metal compounds in the original recipe that contain sulfate were replaced with alternatives without sulfate. Magnesium concentration in this experiment was kept constant at the base medium value of 60 mg l^-1^ (Table 1) by using MgCl_2_∙6H_2_O.

Initial cell density (inoculum size) in the culture flasks was 100 cells ml^-1^ and each treatment concentration was conducted in triplicate. Two complete, independent trials were conducted for the experiment with MgSO_4_ and one trial was conducted with Na_2_SO_4_. Cell density in each flask was determined every 3 days until batch cultures reached late stationary phase (see Analytical procedures section).

#### Effects of salinity under different major salt scenarios

An earlier study showed that abruptly increasing the salinity of modified ASM from 5 to 30 psu by adding NaCl strongly suppresses golden alga growth [7]. The present experiment was designed to determine if growth suppression at high salinity also occurs under a relatively complex salt scenario similar to that of seawater (Table 1). Modified ASM (see preceding section) and Instant Ocean® sea salts (IO) were used to prepare respective experimental media at salinities of 5 and 30. Modified ASM has a salinity of 5, and salinity of 30 in this medium was achieved by simply adding NaCl. In IO, salinities of 5 and 30 were prepared by direct addition of the appropriate amounts of commercial salt preparation to deionized water. Salinity of experimental media was confirmed by measurement with a multiparameter probe. Nominal concentrations of major ions in culture media for this experiment and in seawater are shown in Table 1 [the elemental composition of IO and seawater is based on Atkinson and Bingman [34]].

Culture media for these experiments were filter-sterilized (Nalgene, 0.45 μm, sterile analytical filter unit, Thermo Fisher Scientific Inc., Waltham, MA, USA) after addition of nutrients and trace metals. Filter-sterilization was used instead of autoclaving because the latter procedure caused salts in IO media to precipitate. Other media and culture procedures were as described earlier. All treatments were conducted in triplicate.

#### Effects of differences in sulfate concentration at high salinity

Results of experiments under the preceding sections led to the ad hoc question, can the inhibitory effect of NaCl-dependent high salinity on golden alga growth at 30 psu [7] be reduced or eliminated by a sulfate concentration corresponding to that of seawater at 30 psu? Two trials were conducted in this experiment. The following media were prepared for trial 1: (1) modified ASM at salinity of 5 psu, (2) modified ASM with additional NaCl to raise the salinity to 30 psu, and (3) modified ASM with 2.4 g l^-1^ sulfate (as MgSO_4_) and the appropriate amount of NaCl to achieve a salinity of 30 psu. Media for the second trial were prepared in the same manner except that the source of sulfate to achieve the concentration of 2.4 g l^-1^ was Na_2_SO_4_. The concentration of sulfate in the third treatment of each trial corresponds to its concentration in seawater adjusted to a salinity of 30 psu (Table 1). Media, culture and cell enumeration procedures were as described previously except that treatments for the first trial were conducted in quadruplicate.

#### Effects of changes in fluoride concentration at low salinity

An earlier field study reported that surface waters with a history of golden alga blooms have relatively high concentrations of fluoride [12]. This experiment was designed to determine whether this association is causal and independent of salinity. The formula for modified ASM does not include fluoride. Four nominal fluoride concentrations were prepared for this experiment by adding NaF (Sigma S6776) to base medium: 0, 2.25, 11.30, and 56.50 mg l^-1^. Cell culture and enumeration procedures were as described earlier and all treatments within a trial were conducted in triplicate. Two independent, complete trials were conducted.

### Analytical procedures

#### Cell counts

Cell density was determined as described by Rashel and Patiño [7]. Briefly, aliquots of 500 μl of experimental cultures were taken from each replicate flask at 3-day intervals and used to determine abundance using a hemocytometer under a compound microscope. To maintain the total cell counts below 50,000 cells ml^-1^, sub-samples were diluted with fresh medium when needed. A total of three counts per replicate flask were taken and the average cell number was reported for each replicate.

#### Estimation of growth parameters and statistical analyses

Growth parameters estimated for all experiments were exponential growth rate (*r*, day^-1^) and maximum cell density (cells ml^-1^). In addition, early cell density (cells ml^-1^) was estimated for the sulfate experiments at low salinity (section Effects of changes in sulfate concentration at low salinity) to assess correlations between growth indices with the full range of sulfate concentrations tested as well as global relationships among growth indices. Maximum cell density was the highest cell count achieved by each replicate. Early density is an index of cell status and growth during the transition between the lag and exponential phases and, under certain conditions, can influence maximum growth potential even in the absence of changes in exponential growth rate [7].

Growth rate for golden alga was calculated using the following equation [35]:

$$r=\frac{\ln N_{2}-\ln N_{1}}{t_{2}-t_{1}}$$

where N_1_ and N_2_ are cell densities at times t_1_ and t_2_ (t_2_ > t_1_). For each individual replicate, times were chosen so that they bracket the linear portion of the ln-transformed growth curve. Mean values are reported for all growth parameters.

Most data were analyzed by one-way ANOVA followed by pairwise comparisons using Tukey’s Honest Significant Difference (Tukey’s HSD) test. Two-way ANOVA was used in experiments where two full trials were conducted [sulfate (as MgSO_4_) and fluoride]; if no trial or interaction effects were observed, values from both trials were pooled and mean separations assessed by Tukey’s HSD. Spearman correlation analysis was used to assess the association between growth indices and sulfate concentration, and partial correlation analysis was used to determine associations between maximum density (i.e., growth potential) and the other two growth indices (*r* and early density) over the full range of sulfate concentrations. Family-wise error rate was controlled by adjusting *p*-values according to the Holm-Šídák method [36]. Analysis of variance and mean separations were conducted with SPSS 17.0 (SPSS Inc., Chicago, USA), correlation analyses with Statistica version 13.3 (Tibco Software, Inc., Palo Alto, CA, USA), and Holm-Šídák *p*-value adjustments and graphics with GraphPad Prism 6 (GraphPad Software, Inc., La Jolla, CA, USA).

## Results

### Effects of changes in sulfate concentration at low salinity

#### Sulfate source: MgSO_4_

**Fig 1:**
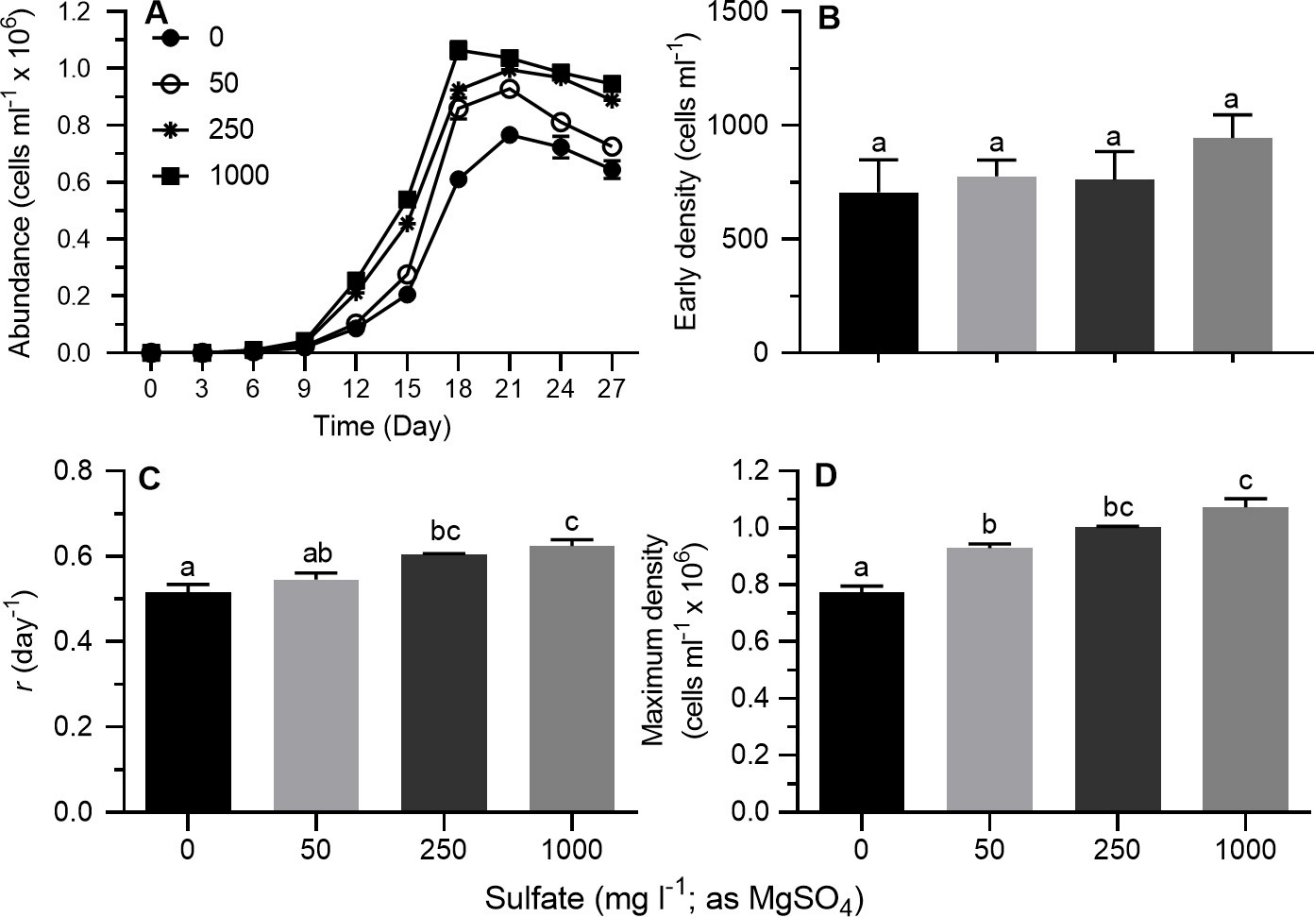
Growth indices of *Prymnesium parvum* as a function of sulfate concentration (0–1000 mg l^-1^, as MgSO_4_) in modified ASM at a salinity of 5 psu. (A) Growth curves, (B) early density, (C) exponential growth rate (*r*), and (D) maximum cell density. Each time point or bar represents the mean (± SEM) of 6 replicates. Bars with the same letter codes do not differ significantly (Tukey’s HSD, *p* < 0.05).

Stationary growth phase was generally achieved on day 21 at all sulfate concentrations (Fig 1A). On a semi-ln plot, the linear portion of the exponential growth phase generally bracketed 3–12 days. Results of 2-way ANOVA showed no differences between trials for *r*, maximum density or early density (Table 2). Sulfate concentration affected *r* and maximum cell density, but not early density (Table 2). No interaction effects were observed between sulfate concentrations and trial for *r*, maximum density or early density (Table 2). Replicates from both trials were therefore pooled for mean separations. As sulfate concentration increased, *r* (Fig 1C; Tukey's HSD, *p* < 0.05) and maximum cell density (Fig 1D; Tukey's HSD, *p* < 0.05) both increased. Early density was 1.34 times higher at 1000 mg l^-1^ compared to 0 mg l^-1^ but ANOVA yielded no significant differences (Fig 1B). Compared to the lowest sulfate concentration, *r* and maximum density at a nominal sulfate concentration of 1000 mg l^-1^ were 1.21 and 1.38 times higher, respectively. Results of Spearman correlation analysis showed that maximum cell density and *r*, but not early density, were correlated with sulfate concentration (Table 3). Result of partial correlation analysis of all data combined indicated that maximum density was associated with *r* but not early density (Table 4).

**Table 2:** Output of one-way and two-way ANOVA of data collected in this study. Growth indices examined in *Prymnesium parvum* cultures included exponential growth rate (*r*), maximum density, and early density. DFn, degrees of freedom numerator; DFd, degrees of freedom denominator; *n*, sample size.

	Response	Effects	DFn, DFd	*F*-value	*p*-value
Effects of changes in sulfate concentration at low salinity (sulfate source: MgSO_4_)	*r*	trial	1, 16	0.205	0.657
	maximum density		1, 16	0.624	0.441
	early density		1, 16	0.000	0.999
	*r*	interaction	3, 16	0.912	0.457
	maximum density		3, 16	0.759	0.533
	early density		3, 16	0.888	0.468
	*r*	concentration	3, 16	9.678	0.001
	maximum density		3, 16	30.94	< 0.0001
	early density		3, 16	0.762	0.532
Effects of changes in sulfate concentration at low salinity (sulfate source: Na_2_SO_4_)	*r*	concentration	3, 8	1.681	0.248
	maximum density		3, 8	0.109	< 0.0001
	early density		3, 8	2.801	0.109
Effects of salinity under different major salt scenarios	*r*	concentration	3, 8	6.006	0.019
	maximum density		3, 8	38.668	< 0.0001
Effects of differences in sulfate concentration at high salinity (Sulfate source: MgSO_4_)	*r*	concentration	2, 9	15.893	< 0.0001
	maximum density		2, 9	81.41	< 0.0001
	Effects of differences in sulfate concentration at high salinity (Sulfate source: Na_2_SO_4_)	*r*	concentration	2, 6	25.995	< 0.0001
maximum density			2, 6	367.39	< 0.0001
Effects of changes in fluoride concentration at low salinity		*r*	trial	1, 16	0.008	0.93
	maximum density		1, 16	2.099	0.167
		*r*	interaction	3, 16	0.846	0.489
	maximum density		3, 16	0.66	0.588
	*r*	concentration	3, 16	20.888	< 0.0001
	maximum density		3, 16	53.882	< 0.0001

**Table 3:** Non-parametric Spearman's correlation of maximum cell density, exponential growth rate (*r*), and early cell density in *Prymnesium parvum* cultures versus sulfate concentration (0–1000 mg l^-1^) in modified ASM at a salinity of 5 psu. Data used for these analyses are those reported in Figs 1 and 2 and Table 4. *n*, sample size.

	Matrix of Simple Correlations						
Source of SO_4_^2-^	Maximum density	*p-*value*	*r*	*p-*value*	Early density	*p-*value*	*n*
MgSO_4_	0.96	0.0005	0.78	0.0005	0.25	0.24	24
Na_2_SO_4_	0.95	0.0005	0.52	0.09	0.68	0.0334	12

*Holm-Šídák adjusted *p*-values for multiple tests within each SO_4_^2-^ source; the adjustment includes tests described in Table 4.

**Table 4:** Pearson partial correlation of maximum cell density with exponential growth rate (*r*) or early cell density in *Prymnesium parvum* cultures as a function of sulfate concentration (0–1000 mg l^-1^) in modified ASM at a salinity of 5 psu. When *r* was the correlation variable, early density was used as a control variable, and vice versa. Data used for these analyses are those reported in Figs 1 and 2 and Table 3. *n*, sample size.

	Partial Correlation with Maximum Cell Density				
Source of SO_4_^2-^	Correlated Variable	Control variable	Pearson *r*	*p*-value*	*n*
MgSO_4_	*r*	Early density	0.76	0.0005	24
	Early density	*r*	0.36	0.17	24
Na_2_SO_4_	*r*	Early density	0.85	0.0024	12
	Early density	*r*	0.94	0.0005	12

*Holm-Šídák adjusted *p*-values for multiple tests within each SO_4_^2-^ source; the adjustment includes tests described in Table 3

#### Sulfate source: Na_2_SO_4_

**Fig 2:**
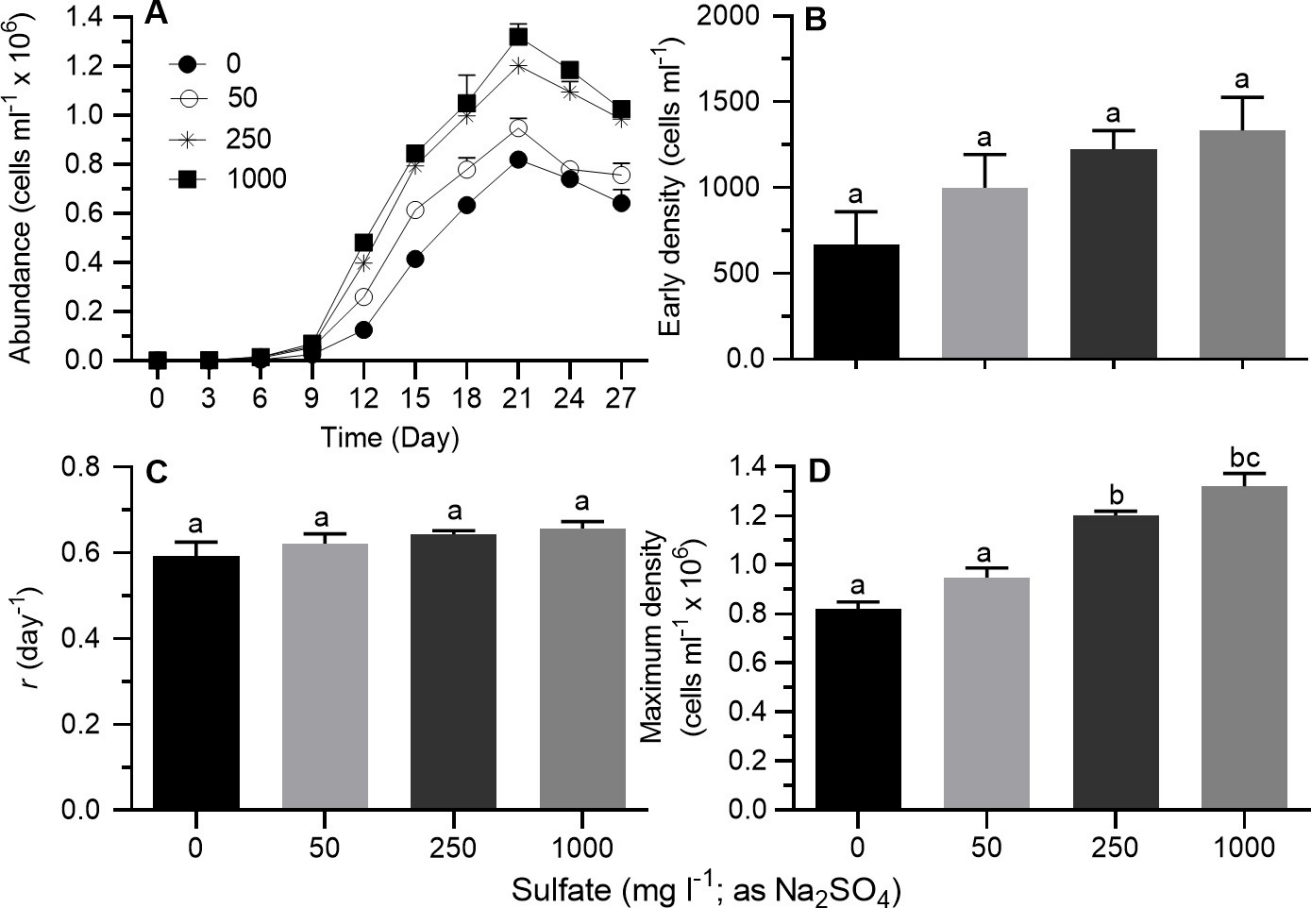
Growth indices of *Prymnesium parvum* as a function of sulfate concentration (0–1000 mg l^-1^, as Na_2_SO_4_) in modified ASM at a salinity of 5 psu. (A) Growth curves, (B) early density, (C) exponential growth rate (*r*), and (D) maximum cell density. Each time point or bar represents the mean (± SEM) of 3 replicates. Bars with the same letter codes do not differ significantly (Tukey’s HSD, *p* < 0.05).

When Na_2_SO_4_ was used as source of sulfate while keeping Mg^2+^ concentration constant, stationary growth phase was generally achieved on day 21–24 at all sulfate concentrations (Fig 2A). On a semi-ln plot, the linear portion of the exponential growth phase generally bracketed 3–12 days. Results of one-way ANOVA showed sulfate concentration had no significant effect on *r* or early cell density but had a significant effect on maximum density (Table 2). As sulfate concentration increased, maximum cell density increased (Fig 2D; Tukey's HSD, *p* < 0.05). Compared to the lowest sulfate concentration (nominal 0 mg l^-1^), maximum density in cultures grown at a nominal sulfate concentration of 1000 mg l^-1^ was 1.61 times higher. Results of Spearman correlation analysis showed that maximum and early cell density, but not *r*, were correlated with sulfate concentration (Table 3). Results of partial correlation analysis, however, indicated that maximum cell density was significantly associated with both, *r* and early density (Table 4).

### Effects of salinity under different major salt scenarios

**Fig 3:**
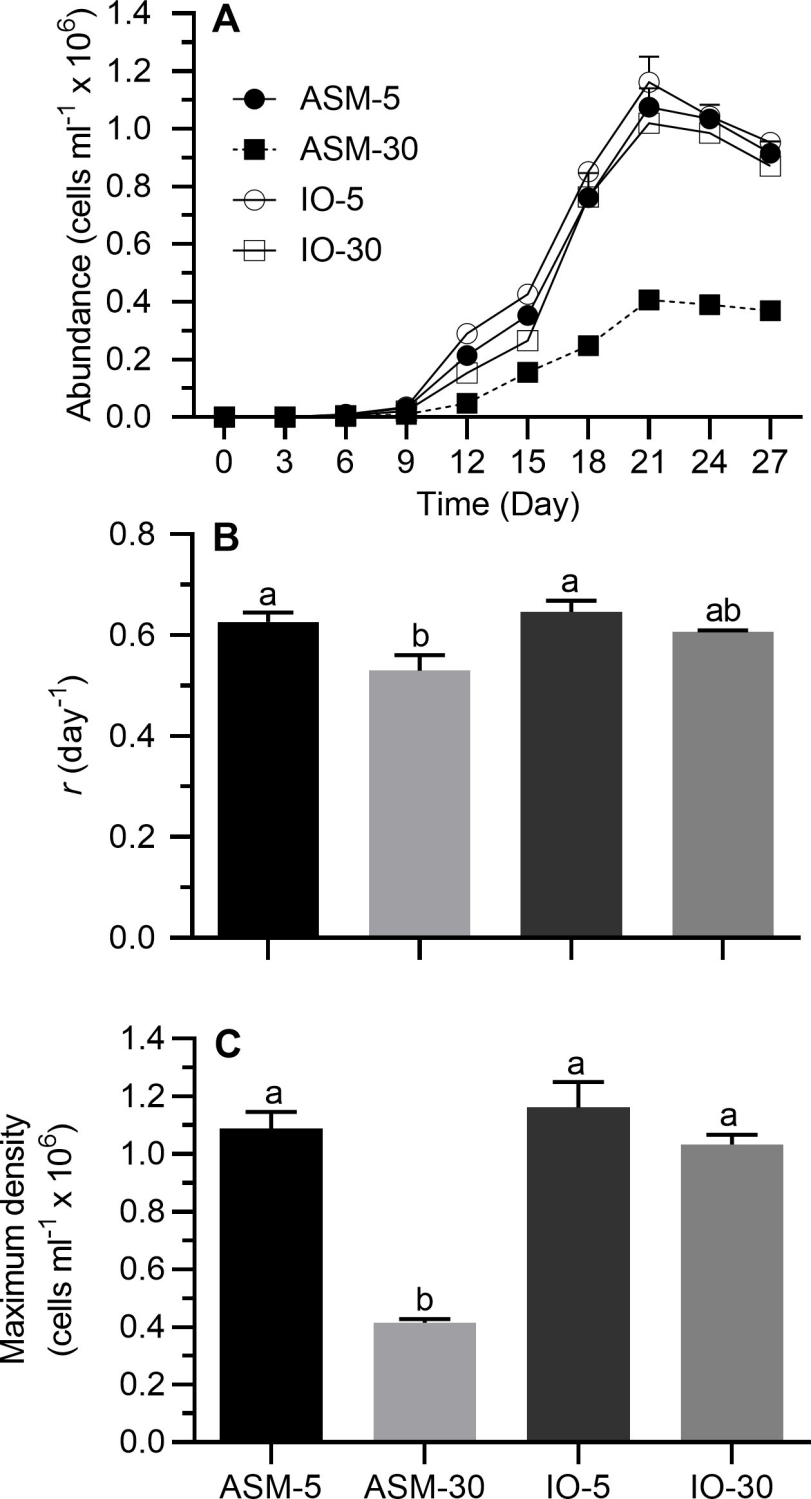
Growth indices of *Prymnesium parvum* as a function of salinity in modified ASM or Instant Ocean with salinities of 5 and 30 psu. Salinity of 30 psu in modified ASM was achieved by the addition of NaCl to 5-psu medium. In IO medium, different salinities were achieved by direct addition of the appropriate amounts of salt mixture. (A) Growth curves, (B) exponential growth rate (*r*), and (C) maximum cell density. Each time point or bar represents the mean (± SEM) of 3 replicates. Bars with the same letter codes do not differ significantly (Tukey’s HSD, *p* < 0.05).

Stationary growth phase was generally achieved on day 21 under all treatment conditions (Fig 3A). On a semi-ln plot, the linear portion of the exponential growth phase generally bracketed 3–12 days. Results of one-way ANOVA showed that treatment had significant effects on *r* and maximum cell density (Table 2). When cells were grown at a salinity of 30 in modified ASM (salinity adjusted by adding NaCl), *r* and maximum cell density were generally lower than in all other treatments (Fig 3; Tukey’s HSD, *p* < 0.05); more specifically, their average values were reduced to 85 and 38 percent of values recorded for *r* and maximum cell density at 5 psu in modified ASM. Growth parameters at 5 or 30 in IO did not differ between themselves or from cells grown at 5 psu in modified ASM (Fig 3B and 3C; Tukey’s HSD, *p* < 0.05).

### Effects of differences in sulfate concentration at high salinity

One notable result of the preceding experiment was that unlike cells cultured in modified ASM at 30 psu, cells cultured in IO at 30 psu did not exhibit growth suppression relative to cells cultured in modified ASM at 5 psu. The primary differences in major ion composition between modified ASM and IO media (at 30 psu) are the higher concentrations in IO media of hardness cations (Ca^2+^ and Mg^2+^), sulfate, and potassium (Table 1). The present experiments evaluated the ability of sulfate to influence growth of golden alga when cells are transferred from 5 to 30 psu (in modified ASM) under relatively high (0.61 g l^-1^; sulfate source, MgSO_4_) or low (0.06 g l^-1^; sulfate source, Na_2_SO_4_) levels of Mg^2+^.

#### Sulfate source: MgSO_4_

**Fig 4:**
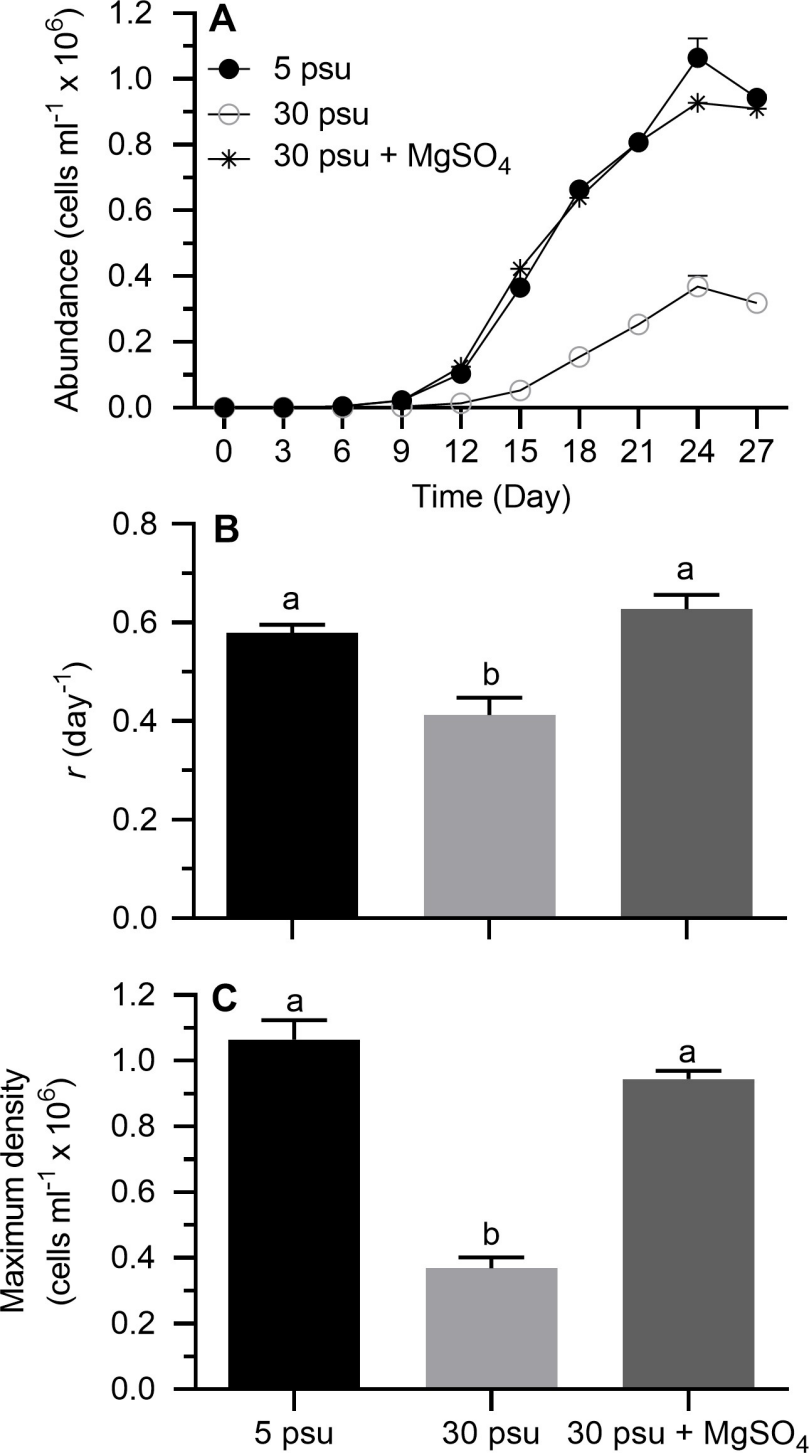
Growth indices of *Prymnesium parvum* in modified ASM as a function of salinity and MgSO_4_ concentration. Salinity of 30 psu in modified ASM was achieved by adding NaCl to 5-psu medium. The treatment receiving MgSO_4_ (2.4 g l^-1^ SO_4_^2-^) received a correspondingly lower amount of NaCl to maintain salinity at 30 psu. (A) Growth curves, (B) exponential growth rate (*r*), and (C) maximum cell density. Each time point or bar represents the mean (± SEM) of 4 replicates. Bars with the same letter codes do not differ significantly (Tukey’s HSD, *p* < 0.05).

Stationary growth phase was generally achieved on day 24 for all treatments (Fig 4A). On a semi-ln plot, the linear portion of the exponential growth phase generally bracketed 3–12 days. Results of one-way ANOVA showed that treatment conditions had significant effects on *r* and maximum density (Table 2). When cells were grown in modified ASM at 30 without the additional sulfate, *r* and maximum density were markedly reduced compared to the other treatments (Fig 4B and 4C; Tukey’s HSD, *p* < 0.05); more specifically, *r* and maximum density in these cultures were reduced to 71 and 35 percent of the values observed in modified ASM at 5 psu. The addition of sulfate (2.4 g l^-1^) as MgSO_4_, however, completely nullified the inhibitory effect of high salinity on golden alga growth (Fig 4B and 4C).

#### Sulfate source: Na_2_SO_4_

**Fig 5:**
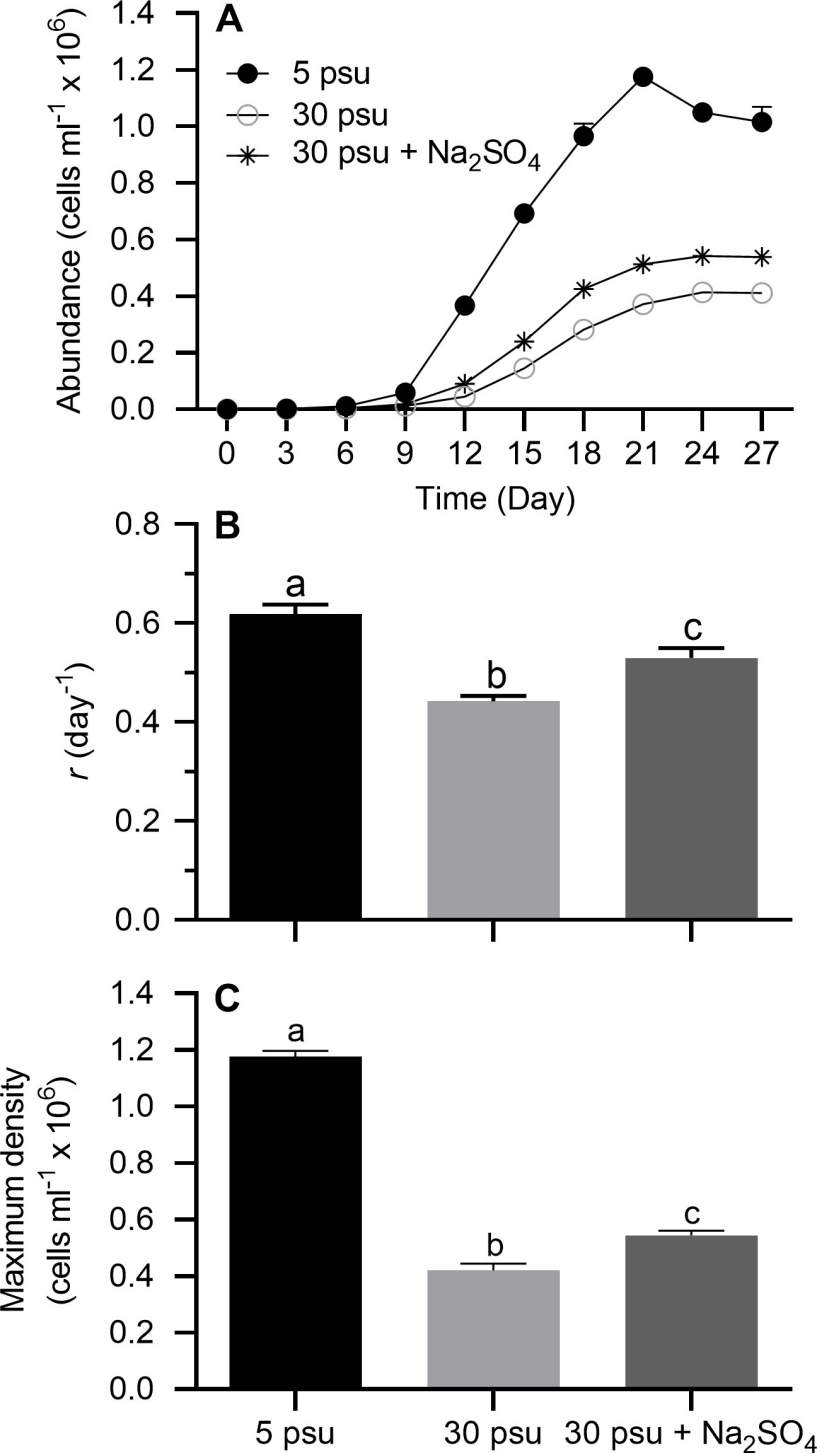
Growth indices of *Prymnesium parvum* in modified ASM as a function of salinity and Na_2_SO_4_ concentration. Salinity of 30 psu in modified ASM was achieved by adding NaCl to 5-psu medium. The treatment receiving Na_2_SO_4_ (2.4 g l^-1^ SO_4_^2-^) received a correspondingly lower amount of NaCl to maintain salinity at 30 psu. (A) Growth curves, (B) exponential growth rate (*r*), and (C) maximum cell density. Each time point or bar represents the mean (± SEM) of 3 replicates. Bars with the same letter codes do not differ significantly (Tukey’s HSD, *p* < 0.05).

Stationary growth phase was generally achieved on day 21–24 under all treatment conditions (Fig 5A). On a semi-ln plot, the linear portion of the exponential growth phase generally bracketed 3–12 days. Results of one-way ANOVA showed treatment conditions had a significant effect on *r* and maximum cell density (Table 2). When cells were grown in modified ASM at 30 without additional sulfate, *r* (Fig 5B) and maximum density (Fig 5C) were significantly reduced compared to control at 5 psu. The addition of a seawater level of sulfate (2.4 g l^-1^) as Na_2_SO_4_ only partially restored growth potential (Tukey's HSD, *p* < 0.05).

### Effects of changes in fluoride concentration at low salinity

**Fig 6:**
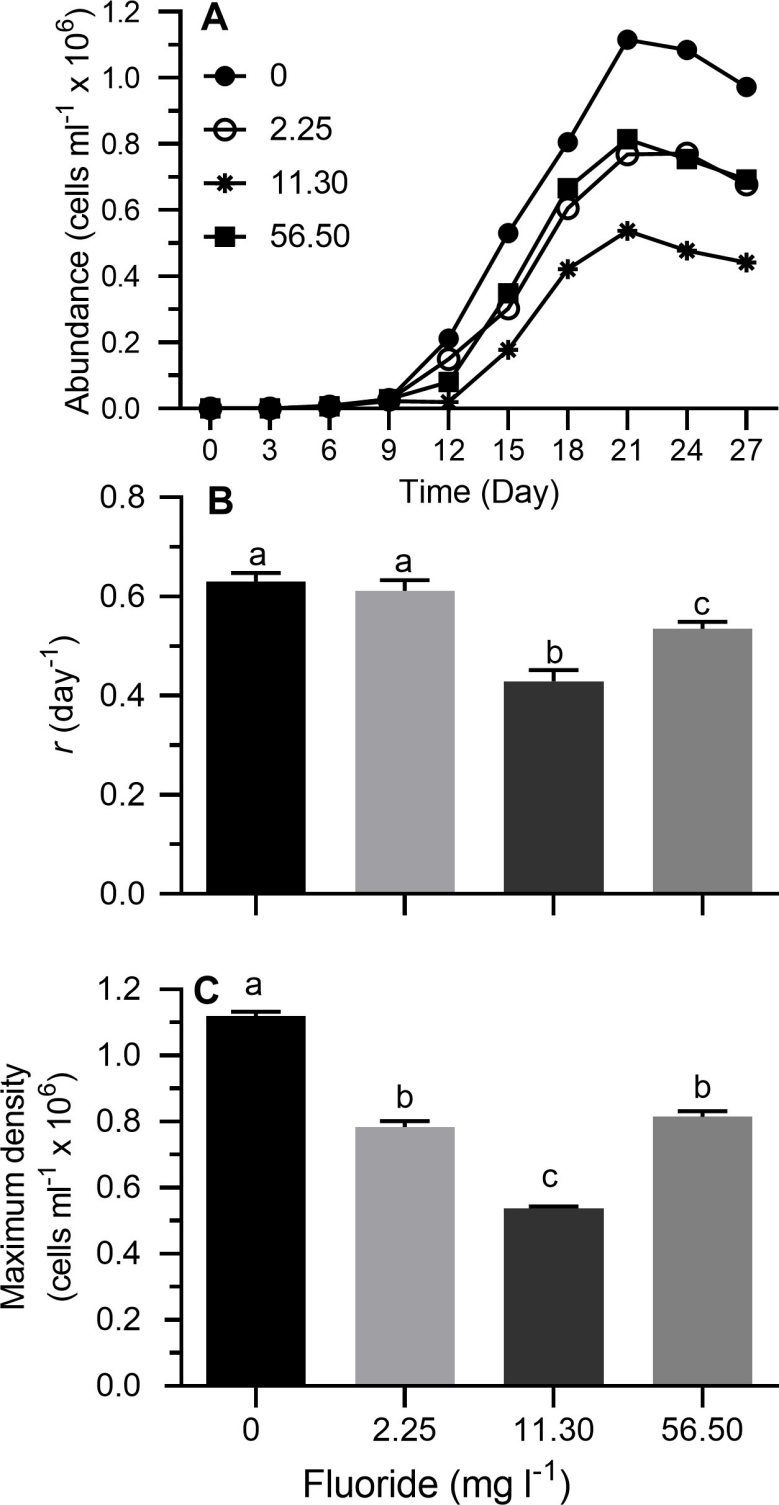
Growth indices of *Prymnesium parvum* as a function of fluoride concentration (0–56.50 mg l^-1^) in modified ASM at salinity of 5 psu. (A) Growth curves, (B) exponential growth rate (*r*), and (C) maximum cell density. Each time point or bar represents the mean (± SEM) of 6 replicates. Bars with the same letter codes do not differ significantly (Tukey’s HSD, *p* < 0.05).

Stationary growth phase was generally achieved on day 21 at all fluoride concentrations (Fig 6A). On a semi-ln plot, the linear portion of the exponential growth phase generally bracketed 3–12 days. Results of 2-way ANOVA showed no differences between trials for *r* or maximum cell density (Table 2). Fluoride concentration affected *r* and maximum cell density (Table 2). No interaction effects were observed between fluoride and trial for *r* and maximum cell density (Table 2). Replicates from both trials were therefore pooled for mean separations. Exponential growth rate decreased as fluoride concentration increased up to 11.30 mg l^-1^, and then increased at the highest concentration tested (relative to growth at 11.30 mg l^-1^) although it was still lower than in the control treatment (Fig 6B; Tukey's HSD, *p* < 0.05). A very similar pattern was observed for maximum cell density; namely, maximum cell density decreased as fluoride concentration increased from 0 to 11.30 mg l^-1^ and then increased at the highest concentration while still remaining below values observed in the control (Fig 6C; Tukey's HSD, *p* < 0.05).

## Discussion

It is well established that growth of golden alga is influenced by salinity under either natural or laboratory conditions [3,4,7,8,11,12,37–40]. Inland field studies of golden alga that included high-salinity sites in their sampling designs, however, reported biphasic associations between abundance and salinity with peak abundance at ~8–12 psu [4–6]. A recent laboratory study where salinity was manipulated by adding NaCl to modified (pre-diluted) artificial seawater experimentally confirmed this biphasic association, with suppression of golden alga growth being particularly notable at > 20 psu [7]. In contrast to the preceding observations, laboratory studies where salinity was manipulated by diluting artificial or natural seawater–which results in the proportional dilution of all ionic constituents–reported what seemed to be log-linear associations between salinity and growth without an apparent inhibition at high salinity [40] or with relatively attenuated biphasic associations [37]. Results of the present study seem to have resolved these conflicting observations by showing that the association between salinity and growth cannot be interpreted without considering the ionic composition of natural waters or artificial media. The present findings also confirmed the working hypothesis that environmentally relevant concentrations of SO_4_^2-^ (in inland habitats) enhance growth of golden alga independently of salinity. Contrary to initial expectations, however, algal growth was negatively influenced by F^-^ at all concentrations tested.

### Effects of sulfate and other ions at low salinity

Maximum density achieved by golden alga cultures at a constant salinity of 5 psu was positively associated with SO_4_^2-^ concentration. While the association was clear regardless of SO_4_^2-^ source, its strength seemed to differ between the two sources; namely, as SO_4_^2-^ concentration increased from nominal 0 to 1000 mg l^-1^, maximum density increased by 61% when the source was Na_2_SO_4_ compared to 28% when the source was MgSO_4_. On the other hand, *r* was positively associated with SO_4_^2-^ only when the source was MgSO_4,_ and early density was associated with SO_4_^2-^ only when the source was Na_2_SO_4_. Results of partial correlation analysis of all data combined, however, revealed that maximum density was strongly associated with *r* regardless of SO_4_^2-^ source, indicating the existence of confounding (masking) effects of early density on this association. Overall, these observations suggest that while Mg^2+^ enhances growth performance and particularly in regard to *r*, SO_4_^2-^ stimulates growth of golden alga in a concentration-dependent manner and independently of salinity. The mechanisms of this association are at present unknown. Sulfate is the primary source of sulfur for algae [19,20], however, and under some conditions can limit productivity in freshwater systems [26]. Thus, the positive association between SO_4_^2-^ concentration and golden alga growth could be interpreted, at least partly, in a nutritional context.

The positive association between Mg^2+^ and golden alga growth revealed by this study may also be of ecological relevance. In brackish waters of the southcentral USA where golden alga blooms have occurred, relatively high levels of SO_4_^2-^ are typically accompanied by correspondingly high levels of hardness cations, including Mg^2+^ [4,12]. Magnesium is a key micronutrient known to influence algal growth, lipid content and composition, and chlorophyll content [33,41–44]. Thus, the relatively high levels of Mg^2+^ present in golden alga inland habitats may also serve to facilitate growth of this species.

### Effects of sulfate and other ions at high salinity

Growth of golden alga was significantly reduced when salinity was abruptly increased from 5 to 30 psu by adding NaCl to modified ASM. This observation confirms an earlier study of golden alga that reported growth inhibition in modified ASM at salinities > 20 psu [7]. When algal cells were transferred to 30 psu in a more complex salt solution (IO) resembling the ionic composition of seawater, however, growth was unaffected. Because Na^+^ and Cl^-^ concentrations are similar in ASM and IO at 30 psu (Table 1), it appears that growth suppression in ASM is the direct consequence of increased salinity and that major ions in IO other than Na^+^ or Cl^-^ prevent or counteract the acute effects of osmotic stress on growth. Sulfate is the only major anion that is present at a higher concentration in IO than ASM at 30 psu (~ 9-fold higher) and among the major cations, Mg^2+^ and Ca^2+^ have the highest relative concentration in IO (~20-fold higher) (Table 1). These three ions–SO_4_^2-^, Mg^2+^, and Ca2+–individually or in combination are likely candidates for the role of osmotic-stress rescue factors.

Adding seawater levels of SO_4_^2-^ (2.4 g l^-1^) using Na_2_SO_4_ as source did not fully prevent growth inhibition caused by raising the salinity from 5 to 30 psu in modified ASM. Maximum cell density and *r* were measurably higher in the presence of the extra SO_4_^2-^ than in its absence but in both cases, growth was still greatly reduced compared to control cultures at 5 psu. When MgSO_4_ was used as sulfate source, however, growth in the high salinity medium was fully restored to control levels. These observations indicate that although SO_4_^2-^ has a minor positive influence on algal growth at high salinity, Mg^2+^ is required for full growth restoration. The nominal concentration of Mg^2+^ in ASM without additional MgSO_4_ is 0.06 g l^-1^, in ASM with additional MgSO_4_ is 0.61 g l^-1^, and in IO (at 30 psu) is 1.26 g l^-1^ (Table 1). Because the growth performance in ASM with additional MgSO_4_ and in IO were both similar to their respective control values, a concentration of ~0.61 g l^-1^ Mg^2+^ seems sufficient to nullify the inhibitory effect of high salinity. The mechanism by which Mg^2+^ rescues golden alga growth from high-salinity stress was not addressed in this study and is unknown. It should be noted, however, that the addition of seawater concentrations of Mg^2+^ to modified ASM at 30 psu simply restored growth to the same level observed in ASM at 5 psu, and that the latter medium contains a 10-fold lower concentration of Mg^2+^ (0.06 g l^-1^, Table 1). Thus, the rescue effect of Mg^2+^ at high salinity is unlikely to represent a classical nutritional function.

Talarski et al. [45] reported that golden alga transferred from 5-psu medium to 30-psu natural seawater underwent major changes in gene expression, with at least 1507 and 1000 transcripts showing up and down regulation, respectively. Some of the differentially expressed transcripts were associated with salinity stress, osmolyte production, or ion transport. Golden alga in the study of Talarski et al. [45] was maintained in 30-psu seawater for several rounds of culture over several months before analysis. Thus, interpretation of the acute effects of high salinity observed in the present study in the context of the results of Talarski et al. [45] should be made with caution. In plants, however, Mg^2+^ is necessary for the proper functioning of hundreds of enzymes [46] and exposure to environmental stress often results in higher demand for this ion in order to maintain energy balance [47]. Elevated Mg^2+^ may thus facilitate the major metabolic readjustments necessary to cope with osmotic stress [45], which in turn may have served to prevent growth inhibition at high salinity in the present study.

### Effects of fluoride

Fluoride concentrations in inland waters typically range from 0.01 to 0.30 mg l^-1^ but natural or anthropogenic inputs can result in much higher concentrations [29,31,48]. Exposure of algae to high F^-^ levels can result in growth suppression although the effective concentration varies widely among species [31,49,50]. Growth suppression may be due, at least partly, to impaired photosynthesis [50]. Growth enhancement after exposure to fluoride also has been observed in some species [31], and enhancement at low concentration followed by suppression at higher concentration (hormesis) has been reported as well [51]. In the few field studies of golden alga where F^-^ concentration was measured, blooms seemed to occur in water bodies with concentrations >1 mg l^-1^ [12,17]. Given the wide variation in algal responses to F^-^, VanLandeghem et al. [12] hypothesized that putative tolerance to F^-^ could favor growth of golden alga, a mixotrophic species, by providing a competitive advantage over phototrophic algae. Results of the present study rejected the hypothesis; namely, growth inhibition occurred at the lowest nominal concentration of F^-^ tested (2.25 mg l^-1^) making golden alga one of the least fluoride-tolerant algal species studied to date. This finding raises the question of why, despite their intolerance to this ion, is golden alga able to thrive in water bodies with relatively high F^-^ concentration. The relatively high levels of F^-^ in golden alga habitat, however, are accompanied by relatively high levels of water hardness [12,17] and as hardness increases, the bioavailability of fluoride ion decreases [31]. Thus, the positive association between F^-^ levels and golden alga presence in the field could simply be a spurious observation.

## Summary and conclusions

This study showed that growth of golden alga at low (brackish) salinity responds positively to elevated SO_4_^2-^ and Mg^2+^. These observations support the hypothesis that spatial differences in SO_4_^2-^ concentration are partly responsible for determining the inland distribution of golden alga [11], and also provide physiological relevance to reports of relatively high Mg^2+^ concentrations in water bodies with a history of golden alga blooms [12]. At high salinity, however, the influence of sulfate on golden alga growth was minor while elevated Mg^2+^ appeared to be necessary to maintain growth under osmotic stress. Growth of golden alga was negatively associated with F^-^ concentration; consequently, positive associations between F^-^ and algal abundance observed in the field [12] may simply be coincidental. Overall, results of the present study highlight the importance of considering the ionic composition of natural waters or experimental media in studies of the association between salinity and golden alga distribution or growth.

Haptophytes belong to the red lineage of algae (Chromista), which traces its origin to the marine environment and which currently dominates marine phytoplankton communities [24]. Sulfur is required for algal growth and can be limiting in freshwater systems but not in the ocean, where its levels are much higher [22,26]. Below normal seawater concentrations, growth of marine chromists (including the haptophyte, *Emiliania huxleyi* Lohmann) associates positively with SO_4_^2-^ concentration while growth of marine cyanobacteria and green lineage species seems to be largely unaffected [22,27]. It has been proposed that the growth response of extant marine chromists to SO_4_^2-^ is a vestigial trait that allowed their ancestral species an advantage over cyanobacteria and green algae as oceanic SO_4_^2-^ concentrations increased during the Mesozoic Era [22,27,52]. In this context, the ability of golden alga to increase its growth in the presence of elevated SO_4_^2-^ (up to at least 1000 mg l^-1^) in brackish environments is intriguing and suggests this response may share the same evolutionary path as that of its marine relatives. Curiously, growth of the freshwater cyanobacterium *Microcystis aeruginosa* Kützing is suppressed at SO_4_^2-^ concentrations as low as 40 mg l^-1^ [53], suggesting elevated sulfate has the opposite effect than in golden alga. In contrast to marine chromists, however, the present results with golden alga indicate that the ability of SO_4_^2-^ to maintain or stimulate growth at high salinity is limited and overshadowed by the importance of Mg^2+^. A mechanistic understanding of the growth responses to SO_4_^2-^, Mg^2+^ and other ions (e.g., Ca^2+^) at environmentally relevant levels and under different salinity scenarios will be necessary to clarify their ecophysiological and evolutionary relevance to golden alga.

## Supporting information

S1 File Raw data. (XLSX)
